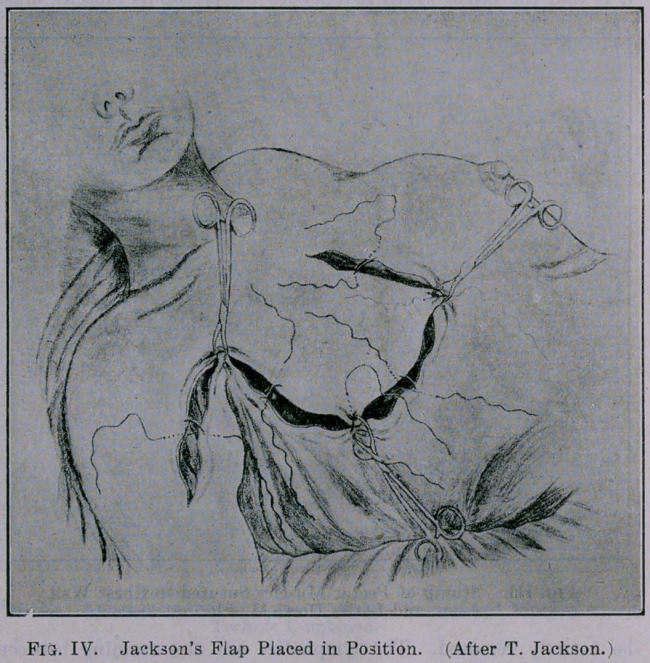# Important Factors to Be Considered in the Diagnosis and Treatment of Carcinomata of the Breast*Read before the Milam County Medical Association, May, 1914.

**Published:** 1914-09

**Authors:** F. C. Floeckinger

**Affiliations:** Taylor, Texas


### TEXAS MEDICAL JOURNAL

Dr. F. E. Daniel, Founder. Established July, 1885

MRS. F. E. DANIEL, - Publisher and Managing Editor

Published Monthly.—Subscription, $1.00 a Year.

VOL. XXX AUSTIN, SEPTEMBER, 1914 NO. 3

The publisher is not responsible for views of contributors

### Original Articles.

For Texas Medical Journal.

### Important Factors to be Considered in the Diagnosis and Treatment of Carcinomata of the Breast.*

BY DR. F. C. FLOECKINGER, TAYLOR, TEXAS.

Attending one of the clinics of Dr. John B. Murphy of Chicago, I heard that distinguished surgeon and teacher say that the end results after removal of the breast for carcinoma are as bad as they were twenty years ago. At the first impulse we may think that this statement is too radical, where we have made so much strife in the advancements of diagnosis, and I mean early diagnosis—and in the execution of the technic.

Why is it now that the end results are so bad? Halstead, Bloodgood, Rodman, Judd, etc., they all are of the same opinion as Murphy is. Therefore we must come to the conclusion that there is something wrong. If we go ahead and think this problem over, we will very quickly come to the solution of this question. There are two causes for this unfortunate outcome. First, the ignorance of the general public as to the importance of consulting a surgeon at once, if the patient observes that there is something wrong with the breast. The second cause is the carelessness of the family physician when examining a patient with a lesion of the breast. The physician may not consider it worth while to go into the history of the case thoroughly, and does not impress on the patient a thorough diagnosis. There we are. There is absolutely no excuse why a proper diagnosis should not be made

*Read before the Milam County Medical Association, May, 1914.

when the patient presents itself to the physician with a lesion of the breast. A superficial examination of the breast may show only a small tumor in the breast, adherent to the overlying skin. The axillary glands may not be enlarged or cannot be felt. May be the tumor has grown very slowly and the physician will make a statement to the patient that the tumor is a benign one, but it would be a good idea to have same removed. There is where the doctor made a big mistake. He does not emphasize strong enough the importance of an immediate operation and an examination of the tissue microscopical. This statement may seem a little too hard on the general practitioner but, nevertheless, it is true. Why is it that if a physician attends to a case of appendicitis he will insist on an immediate consultation with a surgeon and advises an immediate operation? Why not give the unfortunate human the same chance, when afflicted probably with a more horrid disease, which finally will kill the patient? There is where the trouble lies. Ignorance and carelessness in examination.

If a strong plea is made for an immediate thorough microscopical section and a stronger plea for an early complete removal of the breast, then the prognosis would be much better.

Unfortunately, we do not know how early metastases through the lymphatic system take place. Metastases of carcinoma will always take place through the lymphatics and not through the blood stream. Sarcoma metastases will take place through the blood stream, but not carcinoma. Metastases of carcinoma take a systematic course. At first the lymphatics are involved, next the long bones, especially the upper end of the femur. Then the spinal column and then the upper end of the humerus. In large clinics cases will come complaining of pain in the hip and an X-ray examination will show a tumor formation of the bone, and when the breast is examined a tumor will be found, which will prove to be a carcinoma. Everyone understands well that such a case is beyond hope. It is the ignorance which brings this unfortunate state of affairs.

If we study the lymphatic stream of the breast we will find that the metastases will take a regular predilection for certain lymphatic channels. We all know that the axilla is the first one in which metastases takes place. Then comes the second intercostal space near the os sternum, then the sixth intercostal space in the anterior axillary line; next comes the supraclavicular fossa, and the last is through the chain of lymphatics to the umbilicus, and then through the ligamentum teres to the liver. This has been brought cut by Hawdley, and he always advises removal of part of the abdominal wall in his operation for removal of breast in carcinoma. Fig. I. In looking over the literature and studying the reports of large clinics we must come to the conclusion that if the supraclavicular glands are infiltrated, then the operation will unfortunately not do any good. If you look at the diagram here you will plainly see the relationship of the lymphatics to the breast. Besides the regular channels of lymphatics, through which metastases

FIG. I. Lymphatics of the Breast and their Distribution.

take place, there is a large network of lymphatics in the skin of the breast, and involvement of these lymphatics will take place any time, not following a certain rule. Those lymphatics may become involved before ever any involvement of the axillary space takes place. But generally we will find the infiltration of those lymphatics in the skin in the latter stages, when there is no hope for a recovery of the patient and is doomed to die a cancer death. Rodman, in his excellent work, and Herman Kuettner of Breslau, who have had such a large experience in carcinomata of the breast, do come to the same conclusion.

The prognosis of a case of carcinoma of the breast is dependent also on the constitution and the age of the patient. You take those young fat women about thirty-five to forty years of age and the end results will be bad and very bad, because the greatest percentage of those women will die a cancer death. Take a woman of about sixty years of age, who is very lean and slender, with hardly any adipose tissue on the body, and the end results will be much better. And I tell you why. At first the lymphatic channels are not as active any more and therefore the carcinomata cells will have a tendency to localize. And, second, we must consider the type of carcinoma involving the breast. The medullary carcinoma or adenoma carcinoma has a greater tendency for early metastases than the scirrhus type, and a scirrhus carcinoma will generally develop in later years.

The cause of carcinoma of the breast is different from the causes, which produce, or better to say, form a predilection for the formation of carcinoma. So far we do not know the cause of carcinoma; we do not know if same is due to a parasite, a bacterium or due to a cell proliferation. As long as we do not know the cause of carcinoma we are helpless to a great extent, and the statement made at the last meeting of the Cancer Research Committee that so far an early removal of the cancerous tissue of the body is the only hope for eradication. Cherny of Heidelberg, Germany, has made very extensive experiments with radium, the X-ray, and mesothorium, but found that they are not a specific for carcinoma. Early removal, followed with X-ray or radium or mesothorium, will help a great deal to save many from a cancer death. But most prominent pointing out is the statement of early removal. There is a certain type of carcinoma which will have a retrograde tendency and get well. I remember well a case a few years ago of an old man of over sixty who came to me afflicted with an ulcerated lower lip. The submental glands were infiltrated, also the submaxillary and sublingual glands. The history of the case pointed very much to malignancy, and, therefore, I removed a small piece, had my pathologist examine same, sent one specimen to the University of Texas and one specimen to a Dallas pathologist. Both of those men could be depended on for strict ethical knowledge. Report came back, epithelioma of the lower lip with extensive infiltration. Would advise early and thorough removal of all the diseased tissue. My pathologist was of the same opinion. There was a diagnosis made by three men to be depended on. I told the man of the diagnostic findings and told him to come in in a few days and let me remove the carcinoma. The man did not come back to me, and I often wondered what became of him, until about six weeks afterwards I met him on the street, and my first glance was, of course, at his face. There was no scar to be seen, and on looking closer found just a little scar tissue. There was no infiltration of the gland tissue of the neck, so I finally asked him what he had done to get well. He explained that his wife was opposed to an operation and begged him not to undergo the operation. So he shaved off his whiskers and beard completely and took pure carbolic acid and touched the whole ulcerated area with the chemical. In a few days the whole ulcer sloughed out and it began to heal at once with a result that there is not the slightest pathological condition present. Now this case is very instructive. We certainly had to do with a retrogade type of carcinoma, where a stimulus produced some certain reactionary body which destroyed the malignant tumor.

A few years ago Professor Fischera, from the University of Rome, Italy, made some very interesting experiments with the use of embryonic tissue intramuscularis for the cure of carcinoma. He had some good results, and explains that as cancer is due to the unlimited production of embryonic cells, an extract of embryonic cells furnishes the necessary check to the production, and the tumor is removed through the normal process.

Still we are at the same point as we were several years ago. A cure for cancer has not yet been found.

A carcinoma has a tendency to develop in regions which are rich in lymphatics and develop after repeated irritation. Such as carcinoma of the lip we find mostly in smokers. In the gallbladder, where the patient has been afflicted for a long time with a chronic cholecystitis with an impacted stone in the neck of the gallbladder. In the stomach, mostly in the region of the pylorus and first part of the duodenum, due to an ulcer of the pylorus or duodenum. Mayo has, through McCarthy and Wilson, stated that nearly 60 per cent of carcinoma of the pylorus have primarily been cases of ulcer of the pylorus, changing into carcinomata. In the uterus the greatest percentage of cancer formation will take place in the cervix, because most of the lymphatics are located at the cervix and there is a tendency for continued irritation due to an old laceration or chronic infection. In the breast the matter is entirely different. If you study the history of those cases you will always hear the story of some injury, such as a blow to the breast or a fall on the breast—an injury of more than a mild degree so that the patient will notice and tell you about it. One injury to the breast is sufficient to produce cancer A patient may have a simple cyst or adenoma for years in the breast, which is only a benignant tumor. But a moderate severe injury to this breast will change the picture very rapidly and carcinomatous degeneration of the benignant tumor will take place.

I have here on the board put down the classification of tumor of the breast, having copied same from Murphy, as I consider this classification very simple and plain.

CLASSIFICATION.

1. Adenoma: Fibro-Adenoma, Cyst-Adenoma, Adenoma with Carcinoma.
2. Galactophorous Cysts: Simple, Intracystic (Adenoma, Papilloma), Duct Papillomata.
3. Cavernous nevi. Angioma: Lymphatic, Blood.
4. Carcinoma: Acinous Carcinoma, Duct Carcinoma.
5. Sarcoma: Simple (Round Cell, Spindle Cell), Chondro-Sarcoma, Osteo-Sarcoma.
6. Displaced Mammary Tumors in Axilla and Chest Wall.
7. Tumors in the Gland: Hydatic, Lipoma, Chondroma, Myoma, Myxoma, Hypertrophy.
8. Granuloma: Syphilis, Tuberculosis, Actinomycosis, Blastomycosis, Metastatic Pus Infection.

Now as to the diagnosis of a cancer of the breast. At first go into the history of the case very thoroughly and then make your physical examination. A carcinoma of the breast will in the most part be adherent to the overlying skin. The tumor will be hard to the touch, often nodular, and found to be adherent also to the gland structure. It will not be encapsulated, except where there is a carcinomatous degeneration of a benign tumor. A sarcoma will not be nodulated and the type of a scirrhus is the most nodulated type of carcinoma of the breast. A cyst will be somewhat fluctuating, and puncture of the cyst with a trocar will always bring out fluid. You never have this fluid in a primary carcinoma, but if the cyst undergoes carcinomatous degeneration, then you will be able to aspirate fluid. In a case of chronic mastitis you will often find the whole breast riddled with pus and the lymphatics may also be involved in the axillary fossa. If a tumor in the breast has been there for a long time and did not grow, we generally content ourselves that we have to do with a benignant growth. But if this tumor, through the influence of an injury, begins to grow rapidly and gives pain, then there is high time to get busy and find out exactly the nature of the case. In a case of this sort the removal of a very small piece of the tissue will not be sufficient, but the whole tumor should be excised and several sections made from different parts of the tumor, because there may be a beginning of a carcinomatous degeneration in one part of the tumor and the other part may show no malignancy at all. In institutions which have a pathologist right there at the operating room, frozen sections can be made at once and report may be given as to the nature of the tumor to the surgeon in a few minutes. If malignancy is pronounced, remove the whole breast and clean out the axillary space. In cases in which the tumor formation is recent, remove a small piece under local anesthesia and make a section and after receiving the report do the radical operation. If we do follow this rule we will save many a woman from a certain death due to neglect in properly diagnosing the case and neglect of an early operation.

Now as to the treatment of those cases. Complete removal of the breast and extensive dissection of the axillary space is very essential. No matter by what method we remove the breast it is important that the whole mass should be removed in one piece, and always begin at the axilla. If you do this you will not come in contact with any carcinomatous tissue and, therefore, autotransplantation of carcinomatous cells will be avoided. If the operation begins in the axilla, you will be able to do most of your work with a dry gauze sponge and you will need only a few hemostatic forceps and you will have no bloody field. The flap method used is of minor importance and will depend entirely on the involvement of the skin, but don't let yourself be influenced on account of getting a nice cosmetic result to do an incomplete operation. If you do not have enough flap to cover the defect, go ahead and do a Thirsch skin grafting. Always outline the skin incision at first with the scalpel.

In an amputation of the breast the following rules should be kept in mind:

1. Extensive undermining of the skin to remove as much as possible of all the lymphatics in the subcuticular tissue.

2. The complete removal of all the fascia structure covering and enveloping the muscles, because in the rule a carcinoma does not involve muscle tissue, but invariably involves the fascia and the gland structure. Bryan of Guy's Hospital in London, who had such an extensive experience in carcinoma of the breast, states that in all the many years of all cases operated on he saw only one case in which a recurrence did take place in the muscle.

3. Complete removal of all the fat and fascia and lymphatics in the axillary space, and if necessary above the clavicle. Fig. II.

4. The protection of the axillary vessels from cicatrix of the skin to prevent edema of the arm, which is due to constriction

**FIG. II. Amputation of the Breast. Jackson's Flap and Murphy's Technic Combined.**

of the axillary vein. This can be avoided by different procedures. The first one who considered the importance of this procedure was Doyen of Paris, France, who used to insert into the axillary space after amputation of the breast and cleaning out of the axilla a piece of human omentum, which he had stored away in the refrigerator. Such fat tissues have a great vitality, provided no infection takes place. The next method used is the application of Inez Jackson's method of flap formation to cover defect and by closing the incision in such a way as the illustration shows you, obliteration of the axillary space is effected. One of the most ingenious methods is the one adopted by Murphy for many years and having given such good results. The underlying principle is, that carcinomatous involvement of the pectoralis muscle is a curiosity. Therefore he does not remove the pectoralis major and pectoralis minor, but uses the muscles to cover the defect in the axilla and protects the axillary vessels. I have had the opportunity to use this method with and without the Jackson method of flap formation—the method is the ideal one. Of the greatest importance it is that the fascia which covers both the pectoralis muscles must

FIG. III. Stump of Pector Muscles Sutured to Chest Wall and Latiss Dorsi Muscle.

be entirely removed. The lymphatics are also running between those two muscles, and, therefore, complete dissection of this structure is essential. Those two muscles are retracted and when the operation is finished, then the stump of the pectoralis minor muscle is at first sutured to the edge of the latissimus dorsi muscle and to the chest wall in front, and then the pectoralis major is sutured over the pectoralis minor to the chest wall and also to the anterior edge of the latissimus dorsi muscle. By doing this we have completely obliterated the axillary space and have given protection to the axillary vessels. At first clean out the axilla, then extend your incision, which has been marked with the scalpel, undermine the skin flap extensively and remove the whole breast in one piece with the axillary content. There are very few vessels which need ligating. The perforating branches nourishing the pectoralis major muscle and coming from the internal mammary artery are the only larger vessels to be ligated in the proper amputation of the glands. In saving this pectoralis muscle we are able to give a result which permits the woman afterwards to be able to raise her arm over the head and gives her the chance to comb her hair.

FIG. IV. Jackson's Flap Placed in Position. (After T. Jackson.)

Therefore we save a part of the attachment of the pectoralis major muscle to the clavicle, as Judd of the Mayo clinic has advised. Fig. III.

5. The prevention of injury of the long subscapular nerve. If this nerve is injured, the woman will not be able to fasten her clothes behind, which is due to paralysis of the latissimus dorsi muscle. Care also must be taken not to injure the anterior thoracic nerve, which supplies the pectoral muscles. But this is easily avoided, as the nerve can be seen very plainly lying close to the chest wall. It happens sometimes that the flap in the Jackson operation is put on a stretch to such an extent that the flap looks pale. In those cases it is a very good idea to make several stab wounds in the flap to overcome the stasis of the capillary vessels, otherwise you will get necrosis of the flap. Judd, in the Mayo clinic, has used this method very extensively and with good results. Fig. IV. The wound can be closed to suit each individual operator and a drainage is inserted through a stab wound.

In conclusion, I wish to state that to save those unfortunate people from certain death, education to the people in general should be instituted to the fullest extent, and the family physician should use all his diplomacy in impressing the patient as to the necessity of an early operation. If those rules are followed I am certain that the great death rate due to cancer death will be lowered considerable.

## Figures and Tables

**Fig. I. f1:**
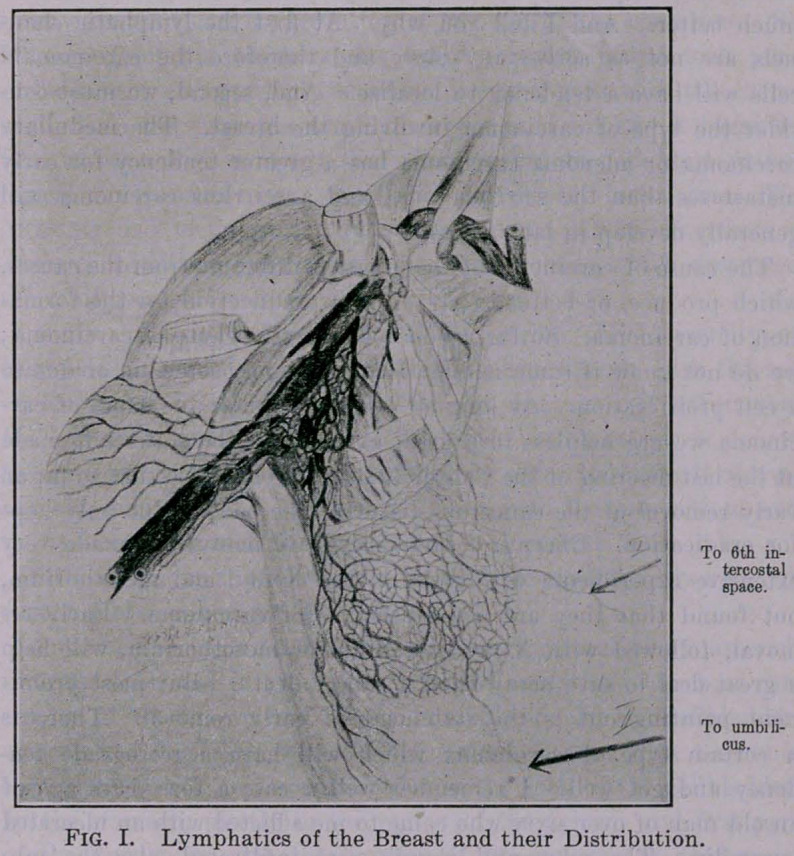


**Fig. II. f2:**
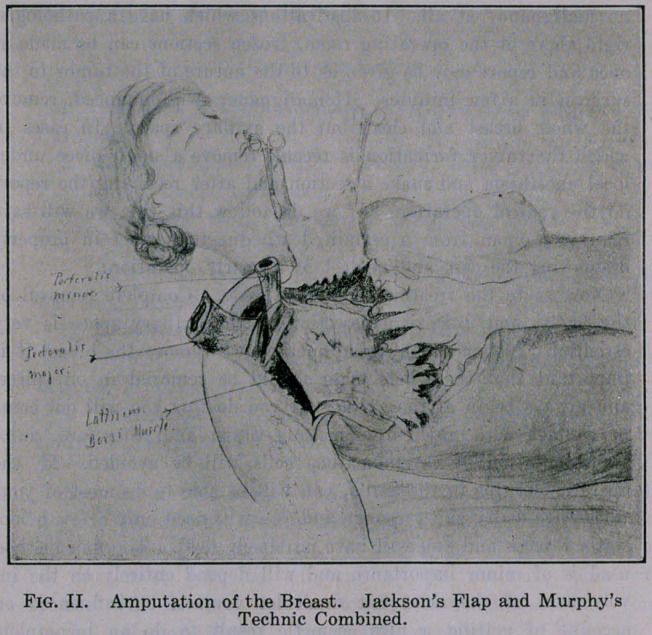


**Fig. III. f3:**
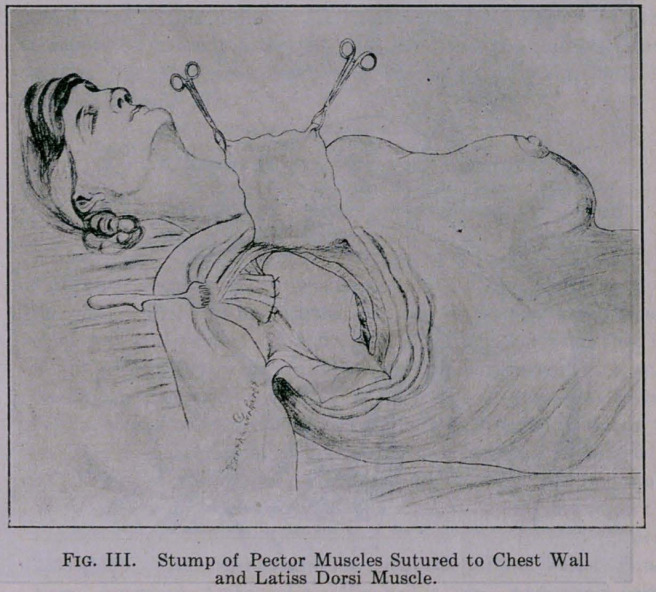


**Fig. IV. f4:**